# Silver nanoparticles as a sustainable approach to enhance crop health and mitigate seawater-induced salt toxicity in chickpea

**DOI:** 10.3389/fpls.2025.1697885

**Published:** 2026-01-16

**Authors:** Nadiyah M. Alabdallah, Salman Latif

**Affiliations:** 1 Department of Biology, College of Science, Imam Abdulrahman Bin Faisal University, Dammam, Saudi Arabia; 2 Basic and Applied Scientific Research Centre, Imam Abdulrahman Bin Faisal University, Dammam, Saudi Arabia; 3 Department of Chemistry, College of Science and Humanities in Al-Kharj, Prince Sattam bin Abdulaziz University, Al-Kharj, Saudi Arabia

**Keywords:** antioxidant, chlorophyll fluorescence, malondialdehyde, root, shoot

## Abstract

Silver nanoparticles (AgNPs) have emerged as promising agents for enhancing plant growth and physiological functions in recent years. However, their role in alleviating salt-induced stress in plants is not yet well understood. In this study, we investigated the impact of foliar sprays of AgNPs1 (300 ppm) and AgNPs2 (400 ppm) on the morphological, physiological, and biochemical responses of chickpea (*Cicer arietinum* L.) seedlings subjected to different levels of salt stress. The chickpea plants were treated with seawater concentrations of 10%, 30%, and 50%, inducing mild, moderate, and severe salt stress. Salt stress significantly inhibited the growth of chickpea, resulting in reductions in both fresh and dry biomass. Additionally, salinity-induced oxidative stress was indicated by elevated malondialdehyde (MDA) levels in chickpea leaves. However, AgNPs, whether applied alone or in combination with salt stress, enhanced several physiological parameters, including chlorophyll content, chlorophyll stability index (CSI), chlorophyll fluorescence (Fv/Fm), and proline levels, while reducing TSS and MDA levels. Moreover, the antioxidant enzyme activity in chickpea leaves improved under salt stress when AgNPs (AgNPs1 and AgNPs2) were applied, suggesting that AgNPs play a key role in mitigating oxidative damage and promoting stress tolerance. Taken together, these results indicate that applying AgNPs can improve the salinity tolerance of chickpea seedlings by enhancing their morphological, physiological, and biochemical responses to salt stress, offering a potential solution for boosting crop yields on salt-affected soils globally.

## 1 Introduction

Global crop production faces numerous challenges due to various environmental factors, with salt stress being one of the most pressing issues (Alluqmani and Alabdallah, 2022; Ulas et al., 2025);. Salt stress severely impairs crop growth and soil function, particularly in arid and semi-arid regions, where poor drainage systems further exacerbate salinity problems (Liu et al., 2026). As a result, salinization has become a significant global concern, affecting an estimated 1,125 million hectares of agricultural land worldwide, posing a serious threat to food security (Hossain, 2019). Salt stress triggers significant disruptions in plant physiological functions, including the loss of membrane stability, excessive accumulation of reactive oxygen species (ROS), reduced photosynthetic performance, narrowed stomatal openings, and impaired antioxidant enzyme activity (Alabdallah and Alluqmani, 2022; Farooq et al., 2024). The production of ROS, such as hydrogen peroxide (H_2_O_2_), MDA, superoxide anions (O_2_^•–^), and hydroxyl radicals (•OH), can trigger oxidative stress within cellular structures. This oxidative stress results in damage to essential biomolecules such as DNA, proteins, lipids, and enzymes, compromising cellular integrity and functionality (Hasan et al., 2021a; Fathi et al., 2025; Jahan et al., 2025). Moreover, the degradation of productive agricultural soils due to salinity limits the use of land and cropping systems, which adversely affects food production and supply in regions dependent on saline-affected areas (Demo et al., 2025). Therefore, implementing affordable and sustainable management practices to support plant morphological, physiological, and biochemical health under salt stress has become essential for ensuring food security in salt-impacted areas.

Saudi Arabia’s arid climate and chronic freshwater scarcity have made soil salinity a major constraint to crop productivity. Agricultural soils in the country frequently exhibit electrical conductivity (EC) values ranging from 4 to 16.4 dS m^-1^, classifying large areas of cultivated land as saline to highly saline (Al-Wabel et al., 2020; Hamed et al., 2024). Although chickpea (*Cicer arietinum* L.) is not yet a dominant crop, interest in its cultivation is increasing under controlled and alternative irrigation systems as part of efforts to promote sustainable legume production in arid regions. Consequently, identifying effective strategies to enhance chickpea tolerance to salinity stress is of particular regional importance for Saudi agriculture, especially under seawater-based or marginal-quality irrigation conditions.

Nanotechnology has emerged as a promising tool in agricultural research, particularly through the development of nanofertilizers and nanopesticides aimed at improving crop productivity and sustainability (Heikal et al., 2020). Nanoparticles have been shown to enhance crop growth, stress resilience, and resource-use efficiency, especially under salt-stressed conditions where conventional agronomic practices often prove inadequate (Taran et al., 2017). The effectiveness of nanoparticle is strongly influenced by their physicochemical properties, including particle type, shape, size (typically 1–100 nm), and applied dosage (Alabdallah et al., 2021; Alabdallah and Hasan, 2021). Among various nanomaterials, AgNPs have received considerable attention due to their reported ability to stimulate plant growth and improve physiological performance under multiple abiotic stresses, including salinity, drought, heat, and flooding (Mustafa et al., 2015; Aziz et al., 2019; Khan et al., 2020). Despite these advances, studies specifically examining the foliar application of AgNPs in chickpea grown under saline or seawater-based irrigation in arid environments remain scarce, representing a critical knowledge gap.

Although AgNPs have demonstrated beneficial effects on plant performance under stress, their safe application in food crops warrants careful evaluation. Previous studies indicate that foliar-applied AgNPs are largely retained within leaf tissues with limited translocation to grains or seeds; however, excessive or prolonged exposure may pose risks of bioaccumulation and environmental contamination (Tripathi et al., 2017; Noga et al., 2023). Therefore, the integration of AgNPs-based strategies into saline agriculture requires a balanced assessment of both agronomic effectiveness and biosafety to ensure environmental sustainability and consumer safety.

Chickpea (*Cicer arietinum* L.) is a significant legume crop globally, known for its richness in proteins and carbohydrates and low fat and ash content. It also serves as a valuable source of minerals, vitamins, amino acids, and unsaturated fats (Jukanti et al., 2012). Despite its nutritional importance, chickpea productivity is increasingly constrained by soil and irrigation-induced salinity, which causes substantial yield losses. Consequently, identifying effective and sustainable strategies to enhance chickpea tolerance to salt stress has become a research priority. AgNPs have emerged as a potential tool for improving crop growth and physiological performance under abiotic stresses, including salinity. Therefore, this study aims to evaluate the effects of different foliar-applied AgNPs doses on the morpho-physiological responses of chickpea seedlings under seawater-induced salinity, focusing on growth and biomass accumulation, photosynthetic pigment content, osmolyte accumulation, lipid peroxidation (MDA), and antioxidant enzyme activities. We hypothesize that foliar application of AgNPs will mitigate the adverse effects of seawater-induced salinity by enhancing plant growth and photosynthetic performance, reducing oxidative damage, and stimulating antioxidant defense mechanisms in chickpea seedlings.

## 2 Materials and methods

### 2.1 Silver nanoparticles biosynthesis and characterization

AgNPs were synthesized using silver nitrate solution as substrate and seeds extract of medicinal plant of African juniper (*Juniperus procera*) as stabilizing and reducing agents. A technique described by Jukanti et al. (2012) was used to prepare the solution. A total of 50 mL of 1 mM silver nitrate solution was mixed with 100 mL of *J. procera* seed extract (1:1 v/v) and followed by heating at 60°C for 30 min till the colour of reaction mixture was changed from light yellow to brown. The characterization of produced nanostructure was done using different analytical approaches; The optical absorption spectrum was estimated in the range of 200–800 nm using UV–Vis spectrophotometer (UV-1800, SHIMADZU, Japan), while the shape and size of AgNPs was characterized using scanning electron microscope (SEM) and Fourier-transform infrared spectroscopy (FTIR) spectra of the AgNPs was recorded using Fourier transmission infrared spectrometer (Perkin Elmer) in the range of 5000–100 cm^−1^.

### 2.2 Growth conditions

The present study was conducted at the Department of Biology, College of Science, Imam Abdulrahman Bin Faisal University, to evaluate the effects of AgNPs on the growth and physiology of chickpea (*Cicer arietinum* cv. Bittle-98). The chickpea seeds were purchased from a company in Saudi Arabia named Altuwaijri. The chickpea seeds were sterilized with 4% sodium hypochlorite before planting. A completely randomized design (CRD) was employed in this experiment. Pots were filled with compost (45.1%), sand (34.2%), silt (34.2%), and clay (22.1%) (38 cm in height and 22 cm in diameter). Each pot contained three uniformly grown seedlings, and each treatment consisted of three pots, which served as biological replicates for statistical analysis. Pots were irrigated twice weekly with tap water until the initiation of salinity treatments.

Salt stress was induced by irrigating the plants with seawater solutions at concentrations of 10%, 30%, and 50%, representing mild, moderate, and severe salt stress, respectively, over a two-week period. Natural seawater collected from the Arabian Gulf (Dammam coast, Saudi Arabia) was filtered and diluted with distilled water to prepare these concentrations. The electrical conductivity (EC) of the irrigation solutions was measured directly in the pots using a portable EC meter and averaged 4.2 ± 0.3, 12.8 ± 0.6, and 21.6 ± 0.9 dS m^-1^ for the 10%, 30%, and 50% seawater treatments, respectively, while the control (0% seawater) showed an EC of 1.6 ± 0.2 dS m^-1^. Pots were not leached during the experimental period in order to simulate realistic salt accumulation under saline irrigation conditions typical of arid environments. The seawater contained approximately 35 g/L total dissolved salts, predominantly composed of NaCl along with other major ions such as Mg^2+^, Ca^2+^, K^+^, SO_4_^2-^, HCO_3_^-^, and trace elements. The concentrations were calculated based on standard seawater ionic composition (Na^+^ ≈ 10.7 g/L, Cl^-^ ≈ 19.5 g/L, Mg²^+^ ≈ 1.4 g/L, SO_4_²^-^ ≈ 2.6 g/L, Ca²^+^ ≈ 0.5 g/L, and K^+^ ≈ 0.3 g/L). AgNPs1 (300 ppm) and AgNPs2 (400 ppm) were applied via foliar spray using a hand-held sprayer to ensure even coverage. AgNP treatments were applied to both control (0% seawater) and salt-stressed plants. Sprays were administered twice weekly during the salinity period using a hand-held sprayer until leaf surfaces were uniformly wetted. Control plants received foliar sprays of distilled water only. In total, the experiment included twelve treatments arranged factorially, consisting of three AgNPs levels (0, 300, and 400 ppm) applied across four seawater salinity levels (0%, 10%, 30%, and 50%).

### 2.3 Growth parameters measurements

Shoot and root lengths were measured on a metric scale and expressed in centimeters (cm). Fresh weights (FW) of shoots and roots were determined using an analytical balance (HR-200) and expressed in grams (g).

### 2.4 Chlorophyll stability index, (%) measurements

To determine the CSI, we applied the Murphy (1962) methodology. The computation of CSI was conducted according to the formula provided below:


$$\text{CSI }\left(\%\right)=1-\frac{\text{ Total Chlorophyll Levels in Heat treated Samples}}{\text{Total chlorophyll of non}-\text{heated sample}}\times 100$$

### 2.5 Chlorophyll content determination

Chlorophyll content extraction from chickpea leaves followed the Arnon (1949) protocol. Once the leaf sample had reached room temperature, 0.25 grams were measured and then ground with 5 mL of an 80% acetone solution to dissolve the chlorophyll pigments. The solution was subjected to centrifugation at 4°C for 10 minutes at a speed of 3000 rpm, ensuring separation of the chlorophyll-rich supernatant. Finally, to quantify chlorophyll a and b, absorbance readings of the supernatant were taken at wavelengths of 663 nm and 645 nm.

### 2.6 Chlorophyll fluorescence measurements

The Li-6800 fluorescence leaf chamber (LI-COR Inc., USA) was used to evaluate Fv/Fm, with the chamber linked to an LI-6800 portable photosynthesis system to support the measurements. The evaluation was conducted after the leaves had undergone an overnight dark adaptation process. The topmost, fully expanded leaves of chickpea plants were chosen the evening before the measurement to determine the dark-adapted fluorescence parameters.

### 2.7 MDA content determination

Following the method of Heath and Packer (1968), the malondialdehyde (MDA) content in leaves was assessed. A sample of 0.5 g of leaves was homogenized in 10 ml of 0.1% TCA solution, and the resulting mixture was centrifuged at 12,000×g for 15 minutes to obtain the supernatant. To initiate the colorimetric reaction, 4.5 ml of 0.5% thiobarbituric acid was added to each milliliter of supernatant, and the mixture was heated at 95°C for 30 minutes. The reaction was then halted by cooling in an ice bath, followed by an additional centrifugation at 10,000×g for 15 minutes. The MDA concentration was calculated using a molar extinction coefficient of 155 mM^-^¹ cm^-^¹, which enabled quantification based on absorbance readings.

### 2.8 Proline content determination

The proline content was determined using an approach derived from Bates et al. (1973). Each treatment fresh leaf samples (1 g) were homogenized in 0.5 ml of 3% (w/v) sulphosalicylic acid with a mortar and pestle to create a consistent solution. The homogenate was then mixed with equal parts glacial acetic acid and ninhydrin, with each component in the final solution totaling 0.2 mL. The mixture was heated in a water bath at 100°C for 30 minutes, after which it was quickly chilled in an ice bath to terminate the reaction. Following cooling, 0.4 mL of toluene was added to the mixture, and absorbance at 520 nm was recorded with a Chemito Spectrascan UV 2600 spectrophotometer.

### 2.9 Measurements of total soluble sugars content

The total soluble sugar concentration in leaves was measured following the Yoshida et al. (1973) method. To start, 0.5 mL of the chickpea leaf extract was pipetted into a test tube, to which 4.5 mL of 85% ethanol was added to help dissolve the sugars. Each test tube was then placed in a cold-water bath to stabilize the temperature and prevent unwanted reactions during the careful addition of 10 mL of anthrone reagent. The anthrone was added slowly to avoid sudden changes in temperature or reaction rates. Following the addition of the reagent, each tube was briefly heated in a boiling water bath to initiate the colorimetric reaction. Immediately after heating, the tubes were transferred to an ice bath to rapidly stop the reaction. After one hour of cooling time, absorbance was recorded at 630 nm to quantify the total soluble sugar content precisely.

### 2.10 Antioxidant enzyme activity

For the extraction of antioxidant enzymes, this study adopted the method described by Mukherjee and Choudhuri (1983). Freshly collected leaf samples (0.5 g) were carefully homogenized in 10 mL of phosphate buffer, adjusted to a pH of 7 to maintain enzyme stability. Following homogenization, the solution was centrifuged at 15,000×g for 10 minutes to separate the supernatant. This supernatant, which contained the antioxidant enzymes, was preserved at 20°C and was utilized for subsequent enzyme activity assessments throughout the study.

### 2.11 Superoxide dismutase activity measurements

The activity of SOD was determined by preparing a 0.2 g sample of leaf material, which was ground in 5 mL of cold phosphate buffer (50 mM, pH 7.8) containing 0.1 mM EDTA and 1% polyvinylpyrrolidone (PVP) to prevent oxidation, as described by Hasan et al. (2020). Following homogenization, the mixture was centrifuged at 11,000×g for 20 minutes at 4°C, and the resulting clear supernatant was utilized for the SOD activity assay. The reaction mixture for the assay included 50 mM phosphate buffer (pH 7.8), 13.0 mM methionine, 10 μM nitroblue tetrazolium (NBT), 0.1 mM EDTA, 0.1 mM riboflavin, and 50 μL of the enzyme extract. Absorbance at 560 nm was recorded using a spectrophotometer (LKB-Biochrom 4050) to follow the reaction. SOD activity was quantified by determining the enzyme concentration required to inhibit NBT reduction by 50%, reflecting its superoxide radical dismutation efficiency.

### 2.12 Catalase activity measurements

To determine CAT activity, the Aebi (1984) technique was employed. A reaction mixture was prepared by mixing 12.5 mM hydrogen peroxide (H_2_O_2_) in 50 mM phosphate buffer, set to a pH of 7.0, with the addition of 50 μL enzyme extract. Upon adding the enzyme extract, the reaction began, and the reduction in absorbance at 240 nm was tracked over 3 minutes using an LKB-Biochrom 4050 spectrophotometer. The rate at which absorbance decreased, indicating the decomposition of H_2_O_2_, was then used to calculate the CAT activity.

### 2.13 Statistical analysis

Using Minitab-17 statistical software, a one-way analysis of variance (ANOVA) was conducted to examine the differences between treatment groups. Each bar in the results reflects the standard deviation (SD) from three independent replicates, providing insight into the data’s variability. Statistically significant differences are marked by distinct letters above each bar, with each letter representing a group that is significantly different from the others.

## 3 Results

**Figure 1:**
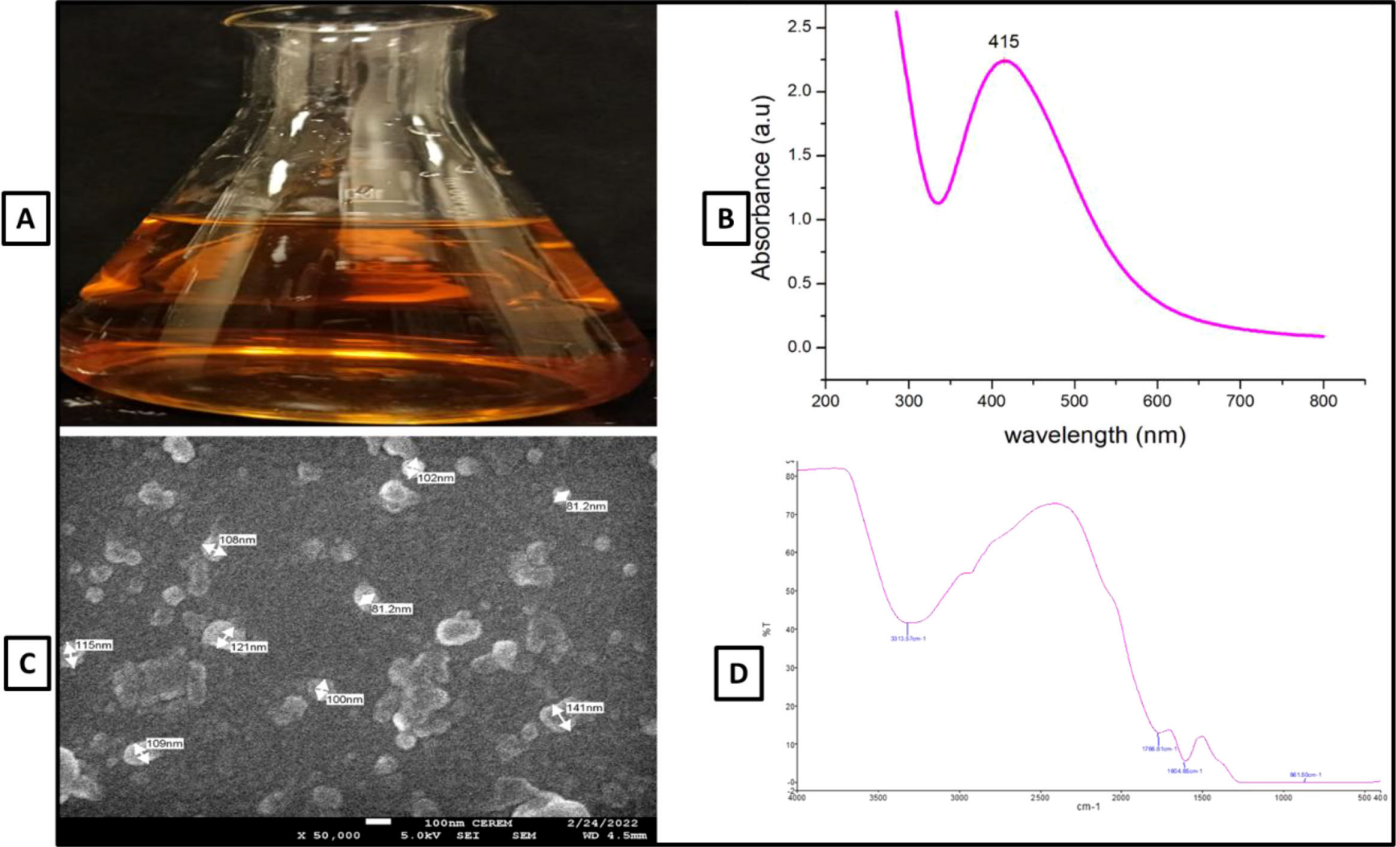
Characterization of AgNPs using different analytical methods; **(A)** colour change showing the reduction Ag NPs **(B)** UV-Visible spectrum of biosynthesized of AgNPs **(C)** SEM image of AgNPs **(D)** FTIR Pattern of AgNPs.

In this study, AgNPs were biologically synthesized using silver nitrate and *Juniperus procera* seed extract, which acted as a reducing and stabilizing agent. The synthesis involved heating the mixture at 60°C for 30 minutes, resulting in a color shift from light yellow to brown, which signified AgNPs formation (**Figure 1A**). UV-visible spectroscopy displayed a distinct absorption peak at 415 nm (**Figure 1B**), and SEM imaging showed that the nanoparticles were spherical, with an average size of 100 nm (**Figure 1C**). FTIR spectroscopy of the biogenic AgNPs revealed characteristic peaks at 331, 1766, 1604, and 861 cm^-^¹, confirming successful synthesis and stability (**Figure 1D**).

**Figure 2:**
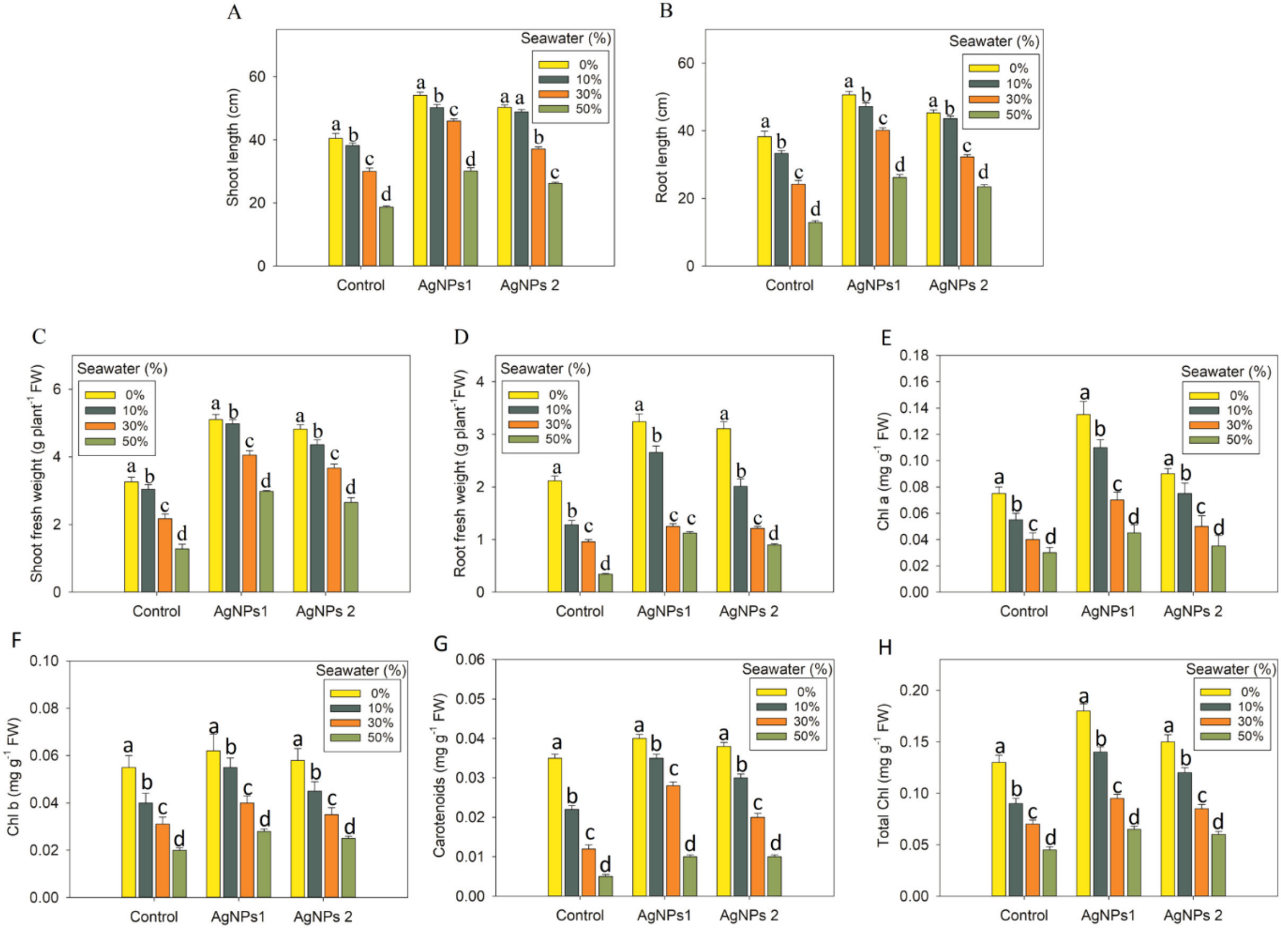
Impact of AgNPs on the **(A)** shoot and **(B)** root length, **(C)** shoot, **(D)** root fresh weight, **(E)** Chl a, **(F)** Chl b, **(G)** carotenoids, and **(H)** total Chl of *Cicer arietinum* seedlings under salt stress. Each bar in the figure represents the standard deviation (SD) calculated from three independent replicates, providing an indication of data variability. Statistically significant differences between treatments are identified by unique letters, with each different letter marking a distinct.

The findings of the study showed reduced growth of the chickpea as well when it was exposed to salt stress. Shoot length, root length, shoot fresh weight, and root fresh weight were reduced by 6%, 26%, 54%; 13%, 37%, 63%; 7%, 33%, 61% and 40%, 54%, 84%, respectively, at the concentration of 10%, 30% and 50% SW over the respective controls (0% seawater). Nonetheless, AgNPs made chickpea grow better and make more fresh biomass in a salt-stressed environment. When AgNPs were added externally, the plants grew much better than those that hadn’t been treated. Chickpea seedlings showed the most improvement when AgNPs1 were used (**Figure 2**).

To examine how AgNPs influence photosynthesis in chickpea leaves, the levels of Chl a, Chl b, and carotenoids were analyzed, providing insight into the impact of AgNPs on these key pigments (**Figure 2**).

**Figure 3:**
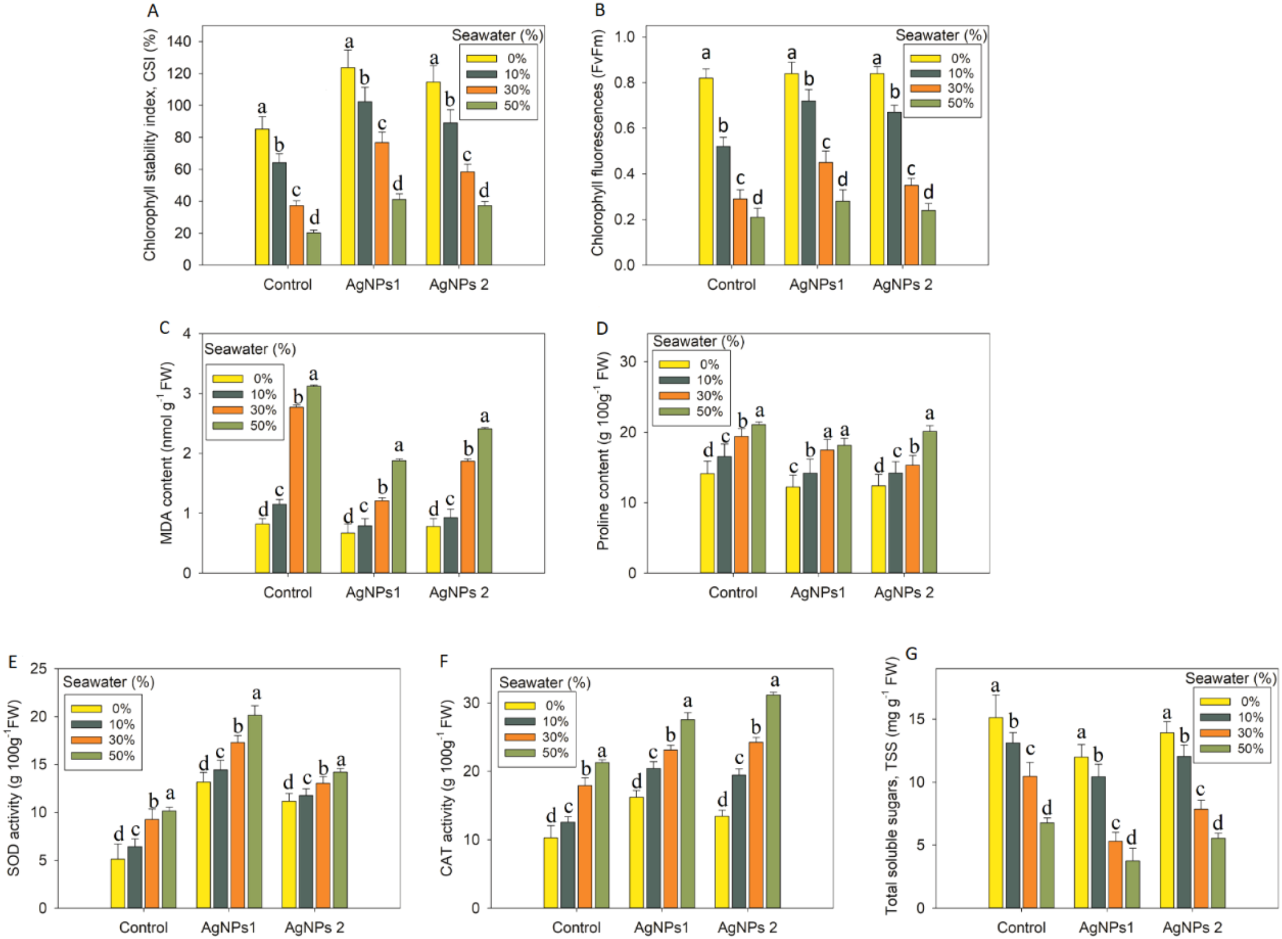
Impact of AgNPs on the **(A)** CSI, **(B)** chlorophyll fluorescence (Fv/Fm), **(C)** MDA, **(D)** proline, **(E)** SOD, **(F)** CAT, and **(G)** total soluble sugars (TSS) content, activities of *Cicer arietinum* seedlings under salt stress. Each bar in the figure represents the standard deviation (SD) calculated from three independent replicates, providing an indication of data variability. Statistically significant differences between treatments are identified by unique letters, with each different letter marking a distinct effect.

Compared to the control seedlings, the SW treatments (10%, 30%, and 50%) significantly (*p* ≤ 0.001) decreased Chl a by 31%, 53%, 63%, Chl b by 25%, 45%, 68%, carotenoids by 38%, 66%, 68%, and total Chl by 28%, 50%, 65%, respectively. Exogenous application of AgNPs1 and AgNPs2, on the other hand, increased Chl a by 27%, 54%,66% and 23%, 39%,59%, Chl b by 23%, 39%, 60% and 14%, 35%, 55%, carotenoids by 4%, 25%,74% and 25%, 42%, 77%, total Chl by 26%, 49%, 65% and 19%, 37%, 57%, respectively, relative to the control seedlings, while chickpea seedlings subjected to 10%, 30% and 50% SW (**Figure 3**). Both higher and lower levels of salt stress decreased the CSI and Fv/Fm in the leaves of chickpea. But the nanoparticles treatments of AgNPs1 and AgNPs2 significantly (*p* ≤ 0.001) increased CSI by 17%, 38%, 67%, and 22%, 49%, 68%, Fv/Fm by 19%, 56%, 79% and 11%, 68%, 80%, respectively, compared to the level in chickpea seedlings that exposed to salt stress only (**Figure 3**).

As shown in **Figure 3C**, the effect of AgNPs was tested by measuring the concentration of MDA to figure out how oxidative damage affects chickpea leaves. The plants that were found to have the highest MDA and proline levels were those that were grown below the seawater level by 50%. At 10%, 30% and 50% SW level, the MDA and proline content increased by 29%,70%, 29% and 15%, 27%, 33%, respectively, over the control (**Figure 3**). In contrast, the application of AgNPs1 and AgNPs2 decreased the MDA content by 15%, 47%, 64% and 16%, 58%, 68%, and proline content by 14%, 30%, 33% and 13%, 19%, 38%, respectively, in chickpea seedlings at 10%, 30% and 50% SW level as compared to control. Furthermore, under 10%, 30%, and 50% SW levels, AgNPs1 and AgNPs2 declined total soluble sugars by 13%, 56%, 69% and 13%, 43%, 60%, compared to that of control. On the other hand, SOD and CAT activities were assessed in chickpea leaves under salinity stress to find out how antioxidant enzymes function properly (**Figure 3FG**). The salt stress boosted SOD and CAT activities at all measured concentrations. Also, the activities of CAT and SOD were significantly boosted by the AgNPs1 and AgNPs2 treatments. At 10%, 30% and 50% SW level, SOD activity was elevated by 9%, 24%, 35% and 5%, 14%, 21%, CAT activity was enhanced by 21%, 30%, 41% and 31%, 45%, 57%, respectively, under AgNPs1 and AgNPs2 treatments, over the respective controls.

## 4 Discussion

Plant growth is significantly reduced under salt stress conditions, mainly because of alterations in the plants water balance.High salt levels in the soil cause osmotic stress, which in turn reduces the soils water potential. This lower water potential prevents the plant from absorbing enough moisture (Hasan et al., 2024). The ability of a plant to tolerate salt stress is also determined by its capacity to maintain growth in areas where water potential is low. In this study, green-synthesized AgNPs have shown promising efficacy in mitigating the adverse effects of salt stress on chickpea plants. When AgNPs were applied exogenously, significant improvements in chickpea growth and biomass were observed (**Figure 1**). These results indicate that AgNPs positively influenced chickpea growth even under saline conditions. The restricted growth often seen in plants under high salinity is typically due to impaired nutrient uptake or enhanced translocation of sodium ions (Na^+^) from root to shoot, reducing the absorption of essential nutrients. This disruption in nutrient uptake reflects the plants stress response to high salinity and its survival mechanisms. However, due to the stress-alleviating properties of AgNPs, they may play a role in enhancing plant growth under these conditions (Almutairi, 2016). Additionally, AgNPs application could be increasing nutrient availability to the plants, supporting growth under stress (Liu and Lal, 2015). Previous research also suggests that AgNPs at various concentrations can enhance seed germination rates and promote plant growth. This effect may be due to the uptake of nanoparticles by seedlings, which supports cell membrane stability, structural integrity, and cellular division (Vishwakarma et al., 2017).

Similar to our observations, AgNPs have been shown to promote plant growth over time and boost tolerance to high salinity by enhancing both growth and physiological processes. Kruszka et al. (2020) reported that AgNPs can upregulate genes responsible for nitrogen recirculation, resulting in improved photosynthetic performance. This boost in photosynthesis is associated with increased production of photo-assimilates, ultimately supporting better plant development (Abdel-Aziz and Rizwan, 2019). In our study, AgNPs treatment showed a positive correlation between chlorophyll content and dry mass accumulation, a finding that could enhance the accuracy of management practices. Chlorophyll content also correlates closely with Fv/Fm (Jahan et al., 2021), suggesting that increased Fv/Fm levels may be linked to higher chlorophyll levels. Consequently, AgNPs have been found to improve PS-II quantum efficiency (Sharma et al., 2012) and enhance fluorescence quenching efficiency (Queiroz et al., 2016). Additionally, the excessive accumulation of Na+ ions in the cytoplasm can inhibit the uptake of other essential ions, thereby affecting multiple metabolic pathways.

**Figure 4:**
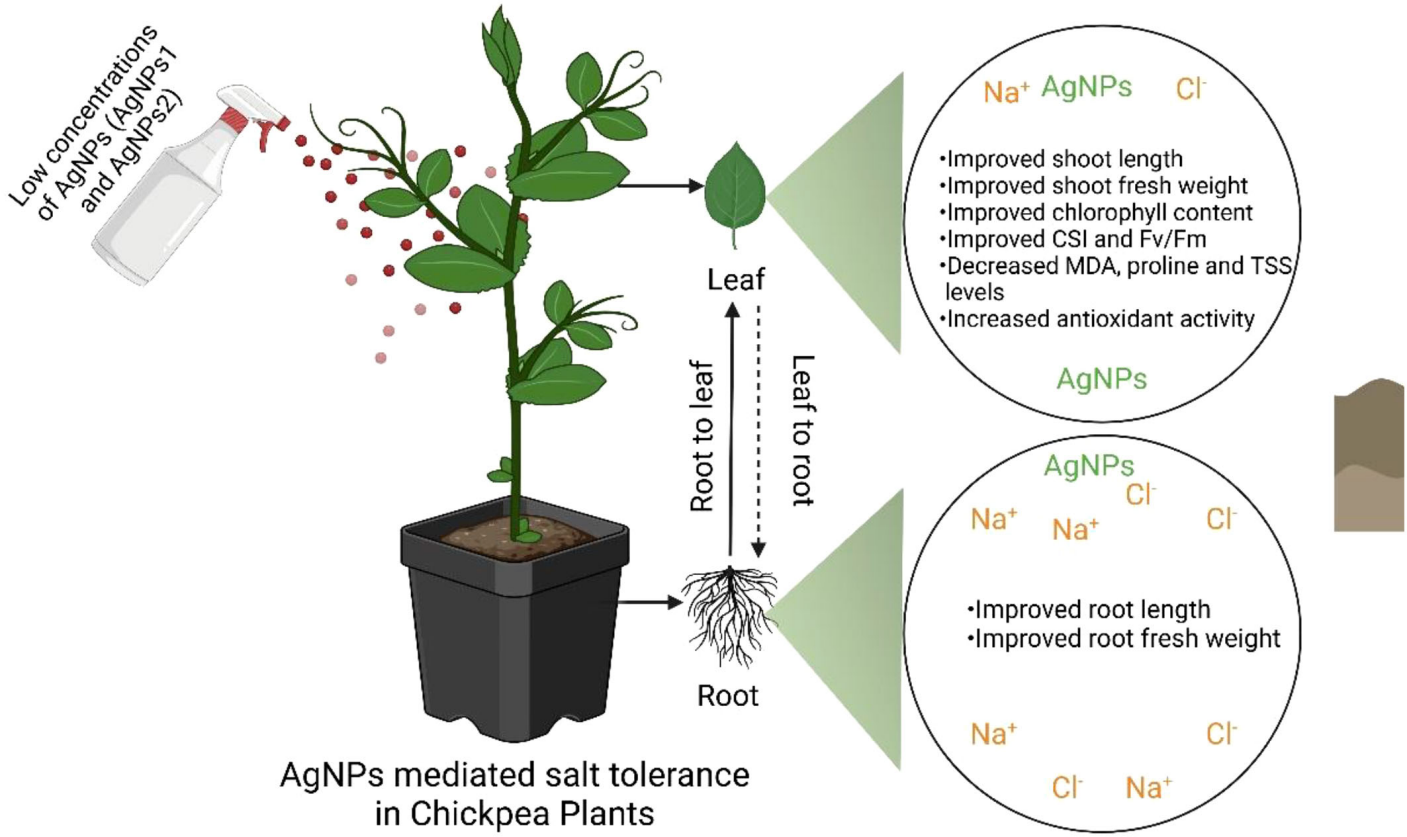
Effect of AgNPs (AgNPs1 and AgNPs2) on enhancing salt tolerance in chickpea plants. AgNPs help alleviate salt stress in chickpea leaves by increasing shoot length, shoot fresh weight, chlorophyll content, and indices for chlorophyll stability (CSI) and fluorescence (Fv/Fm). They also reduce malondialdehyde (MDA), proline, and total soluble sugar (TSS) levels, while increasing antioxidant activity. In the root system, AgNPs contribute to improved root length and fresh weight, supporting greater tolerance to salt stress. This model figure shows the directional transport of Na^+^ and Cl^-^ ions (from root to leaf and vice versa), emphasizing the significant role of AgNPs in promoting growth and salt tolerance in chickpea plants.

Yang and Guo (2018) explain that potassium ions (K^+^) are crucial for the catalytic function of several enzymes; therefore, the presence of sodium ions (Na^+^) disrupts nutritional balance and promotes the generation of reactive oxygen species (ROS) and malondialdehyde (MDA). Similar to our findings, chickpea plants subjected to salt stress exhibited significantly increased levels of MDA and proline, while their total soluble sugars (TSS) decreased when treated with AgNPs (**Figure 4**). The substantial decrease in MDA and proline concentrations observed after AgNPs application may reflect the repair of cellular damage and maintenance of membrane integrity in high-salinity environments (Mir et al., 2018; Mohamed et al., 2017). However, our results also showed that AgNPs considerably reduced TSS accumulation, suggesting a potential role of TSS in cell membrane stability.

In our study, we observed a significant increase in biochemical stress markers, particularly enzymatic antioxidants, in response to salinity stress induced by NaCl. The green-synthesized nanoparticles appear to have a beneficial effect on the plant’s antioxidant defense systems, reducing oxidative stress and supporting overall plant growth (Zaeem et al., 2020). Recent research indicates that the application of nanoparticles can enhance antioxidant enzyme activity across various plant species (Faizan et al., 2018; Hussain et al., 2018; Ali et al., 2019). Under stress conditions, key enzymatic antioxidants, such as SOD and CAT, function as primary defense mechanisms (Abdulmajeed et al., 2021; Hasan et al., 2021b, Hasan et al., 2021c). In wheat, for instance, AgNPs were shown to increase SOD and CAT activity (Abdel-Aziz and Rizwan, 2019), which aligns with our current findings. These results suggest that lower concentrations of AgNPs are particularly effective in enhancing salt tolerance in plants (**Figure 4**).

In addition, while the foliar application of AgNPs effectively mitigated salt stress, potential accumulation in edible parts warrants attention. Previous studies suggest that most foliar-applied nanoparticles remain localized on the leaf surface or within epidermal tissues, limiting their movement to seeds or grains (Tripathi et al., 2017). The concentrations used in this study were relatively low and intended for stress alleviation rather than inducing accumulation. Nevertheless, further studies are recommended to quantify AgNPs residues in harvested chickpeas and evaluate any long-term implications for food safety and human health.

Mechanistically, the foliar application of AgNPs likely alleviates salinity stress through several interconnected pathways. First, AgNPs can penetrate the leaf cuticle and enter cells via stomatal openings or endocytosis, enhancing nutrient translocation and ion homeostasis by regulating Na^+^/K^+^ balance and reducing Na^+^ accumulation in the cytosol. Second, AgNPs act as signaling modulators that induce mild oxidative stress, thereby triggering systemic acquired tolerance and upregulating antioxidant enzyme genes (SOD, CAT, and POD). Third, the nanoparticles may facilitate the synthesis of osmoprotectants such as proline and glycine betaine, maintaining osmotic adjustment and stabilizing proteins and membranes under saline conditions. Finally, AgNPs enhance chloroplast integrity and PS-II stability, resulting in improved photosynthetic efficiency and energy production, which collectively mitigate the negative effects of salt-induced osmotic and ionic stress. Overall, these findings indicate that green-synthesized AgNPs enhance salt tolerance in chickpea by improving physiological stability, maintaining ionic and osmotic balance, and activating antioxidant defense pathways, thus promoting sustained growth and productivity under saline conditions.

## Data Availability

The original contributions presented in the study are included in the article/supplementary material. Further inquiries can be directed to the corresponding author.
